# Arthroscopic scaphocapitate fusion without bone graft; clinical and radiological outcomes

**DOI:** 10.1007/s00264-024-06381-4

**Published:** 2024-11-25

**Authors:** Sherif Ghoneim, Raafat Kamal, Ahmed Semaya, Mohammad Hasan

**Affiliations:** Department of Orthopedic Surgery, Hadara University Hospital, University of Alexandria, Alexandria, Egypt

**Keywords:** Fusion, Arthroscopy, Kienböck’s disease, Arthroscopic scaphocapitate, Wrist

## Abstract

**Purpose:**

Scaphocapitate fusion (SCF) is an important surgical option for carpal pathologies, which are difficult to manage as Kienböck's disease. With the advantages of arthroscopy combined with percutaneous fixation techniques, arthroscopic scaphocapitate fusion can have the best outcome for the patient from a functional perspective. This study aims to evaluate the clinical, radiological, and functional results of arthroscopic SCF.

**Methods:**

The study included thirty patients with stage IIIB and IIIC Kienböck's disease. The articular surfaces were prepared using arthroscopic burr then fixed by Herbert screw. The mean follows up period was about 29 months.

**Results:**

SCF was achieved in approximately seven weeks. There was a statistically significant difference in pre- and post-operative grip strength and Mayo wrist score.

**Conclusion:**

According to our study findings, arthroscopic SCF may be performed with significant improvements and satisfactory clinical and functional results in patients with stage IIIB and IIIC Kienböck’s disease.

## Introduction

The wrist joint is one of the most complex joints in the body, allowing for stability and mobility making most of our daily activities possible [1]. Kienböck’s disease or lunatomalacia is avascular necrosis of the lunate, which is progressive, debilitating, and can lead to chronic wrist pain with significant dysfunction. While Dr. Robert Kienböck, a radiologist in Vienna, described it more than 110 years ago, the exact aetiology is still not clear, nevertheless mechanical, traumatic, and vascular factors have been involved [2].

Numerous treatment modalities for Kienböck’s disease were described. Optimal planning for the treatment modality of Kienböck’s disease depends on the staging, clinical evaluation, and the patient’s expectations [3]. The goals of treatment in order to achieve good results in fusion procedures include solid fusion; if not achieved the results are mostly bad [4]. Lichtman classification, a modification of Stahl’s classification, remains commonly used for staging, provides a useful algorithm in predicting disease progression, and is used as a tool for surgical treatment decisions [3, 5].

SCF materializes to be an important surgical modality for wrist pathologies. Despite the fact that it is a salvage procedure, it preserves joint motion, improves function, and reduces wrist pain [4]. Open partial wrist fusion is infamous for post-operative pain and decreasing range of motion (ROM) because of the open capsulotomy, sectioning important ligamentous elements in carpal biomechanics [6, 7]. This could cause an iatrogenic stiff joint, on top of that already generated by fusion.

Arthroscopy has already shown its advantage in preserving joint mobility compared with open procedures; because the effect of extra-articular adhesion associated with open surgery can be minimized [8]. Moreover, during the first steps of the arthroscopy, a precise evaluation of the damage preceding the procedure is done. Lastly, a cosmetic advantage is associated with the minimally invasive procedure [9].

Combining arthroscopy with percutaneous fixation techniques, arthroscopic SCF can potentially generate the best outcome for the patient [8]. In our study, we evaluate the results of arthroscopic SCF that can be adopted into the standard orthopaedic practice.

## Materials and methods

Arthroscopic SCF was performed on 30 consecutive patients in this prospective case series study. This was a single-centre study between November 2020 and December 2021. A minimum sample size of twenty five patients was calculated using G power at 95% level of significance, p value < 0.05, and power of 0.95. The procedures were performed by a single senior arthroscopic hand surgeon. Inclusion criteria included Lichtman stage IIIB and IIIC Kienböck’s disease (Table 1). Exclusion criteria were active infection of the joint and active proliferative phase of rapidly progressive inflammatory arthritis such as rheumatoid arthritis or crystal deposition disease as in gout. Chronic smoking may affect the rate of union of the fusion, but we have not excluded any patient for such a reason and explained to smoking patients that they have a higher risk of inferior or slower bone fusion.

**Table 1:** Lichtman’s classification [10, 11]

Stage	Radiographs	MRI
0	Intermittent ischaemia	
I	Normal Marrow changes without radiographic changes	T1 signal: decreased T2 signal: variable
II	Increased density Osteonecrosis; no lunate collapse	T1 signal: decreased T2 signal: variable
IIIA	Lunate collapse Radioscaphoid angle < 60◦	T1 signal: decreased T2 signal: variable
IIIB	Lunate collapse with scaphoid palmar flexion Radioscaphoid angle > 60◦	T1 signal: decreased T2 signal: variable
IIIC	Coronal lunate fracture	T1 signal: decreased T2 signal: variable
IV	Radiocarpal or midcarpal degenerative arthritis	T1 signal: decreased T2 signal: variable

Pre-operatively all patients were subjected to history taking, physical examination, and imaging assessment which included plain X-ray of the wrist, computed tomography (CT) scan, and magnetic resonance imagining (MRI).

### Study population

The study population comprised of ten males and 20 females. The mean age was 32.3 years, ranging from a minimum (Min.) of 17 to a maximum (Max.) of 60 years. The dominant side was affected in 21 patients (Table 2).

**Table 2:** Patients Demographic Data (n = 30)

	No. (%)
Age (years)	
Min. – Max	17 – 60
Mean ± SD	32.30 ± 12.15
Median (IQR)	29 (22 – 40)
Sex	
Male	10 (33.3%)
Female	20 (66.7%)
Side	
Right	20 (66.7%)
Left	10 (33.3%)
Dominant hand	
Dominant	21 (70.0%)
Non dominant	9 (30.0%)

SD: **Standard deviation**, IQR: **Inter quartile range**

### Surgical technique

**Fig. 1:**
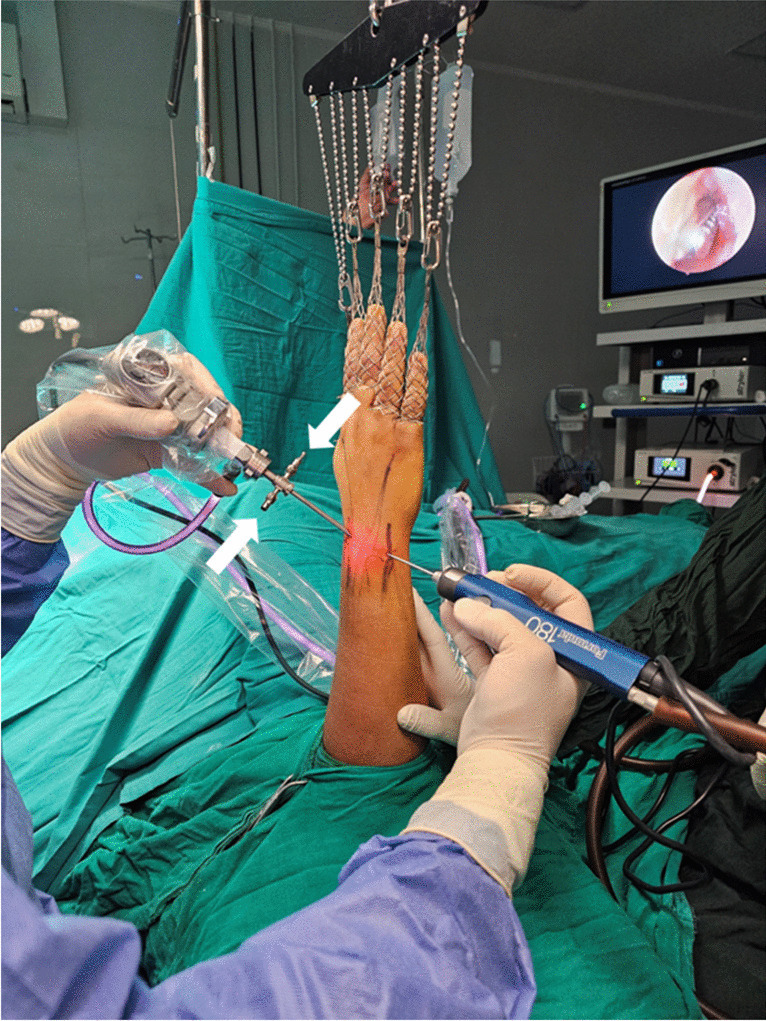
Patient position and wrist traction system. The valve of the sheath should remain open [white arrows], note that no saline was attached to the sheath

In this method, all procedures were performed under general anaesthesia. The patients were positioned supine on the surgical table while the operated arm was supported on a hand table, to which the arm was tied. An arm tourniquet was applied and inflated after disinfection and draping. The wrist was suspended in the wrist traction tower with six to seven kg of traction (Fig. 1).

The procedure started by creating the portals, which are radially 3–4 and radial midcarpal (RMC) portals, and ulnarly 6-R and ulnar midcarpal (UMC) portals. The procedure was performed using the dry technique. The valve of the sheath of the arthroscope should remain open at all times to allow air to circulate freely inside the joint (Fig. 1). Paradoxical suction is necessary to clear the field, or to get the synovium closer to the tip of the shaver.

A routine inspection of both radiocarpal and midcarpal joints was done using a 2.7 mm / 30º angle scope together with a two-valve trocar cannula, and arthroscopic surveillance for final staging of the disease. Radioscaphoid articulation was evaluated to ensure there were no signs of osteoarthritis. Synovectomy was performed as needed.

**Fig. 2:**
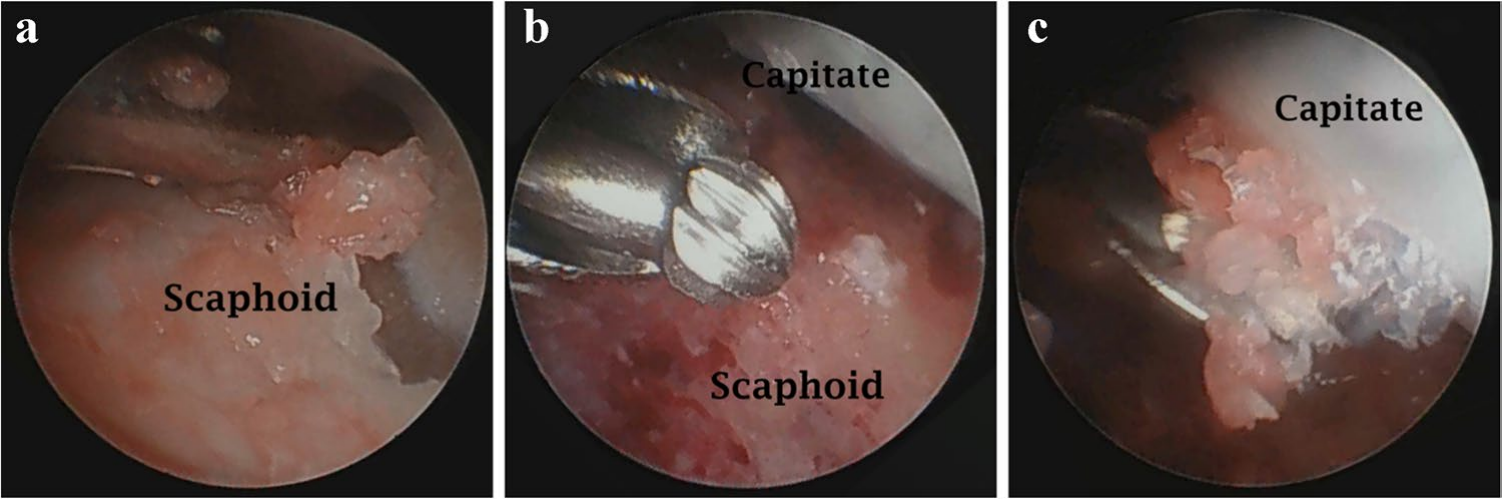
View from ulnar midcarpal portal for the process of preparation of scaphocapitate articulation surfaces. **a**) The articular cartilage of the scaphoid being removed by arthroscopic burr. **b**) The scaphoid cartilage and subchondral bone were removed. **c**) The articular cartilage of the capitate being removed

The articular surfaces of the scaphocapitate articulation which will be fused were then prepared, utilizing radial midcarpal and ulnar midcarpal portals. The cartilage and subchondral bone were removed using a three mm arthroscopic burr until the bleeding cancellous bone was exposed (Fig. 2). Switching the viewing and working portals between radial and ulnar midcarpal portals was very important to ensure that the whole surface of the fusion was well prepared without missing any part that could affect the fusion.

**Fig. 3:**
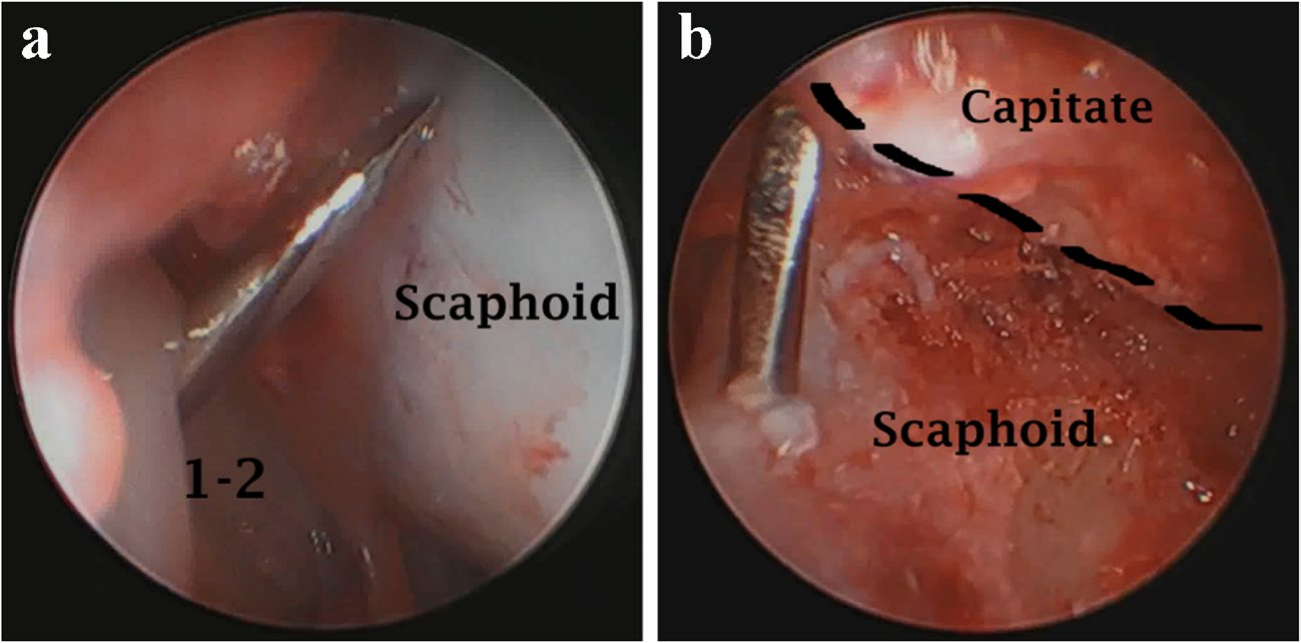
Arthroscopic guided percutaneous insertion of the guide wire for the headless screw. **a**) View from 3–4 portal of scaphoid at radioscaphoid articulation for the process of insertion of the guide wire, where the insertion point is checked. The guide wire was inserted from 1–2 portal. **b**) View from the ulnar midcarpal portal of the guide wire at its exit from the scaphoid. Note that the articular cartilages of the scaphoid and capitate were removed completely and the bleeding cancellous bone was exposed

After the joint surfaces were properly prepared, the appropriate position was maintained with a guide wire for the headless compression (Herbert) screw. The precise location of the insertion point and insertion angle was arthroscopically guided and checked by an image intensifier (Fig. 3). Bone graft was not used in this study, and lunate was not excised.

**Fig. 4:**
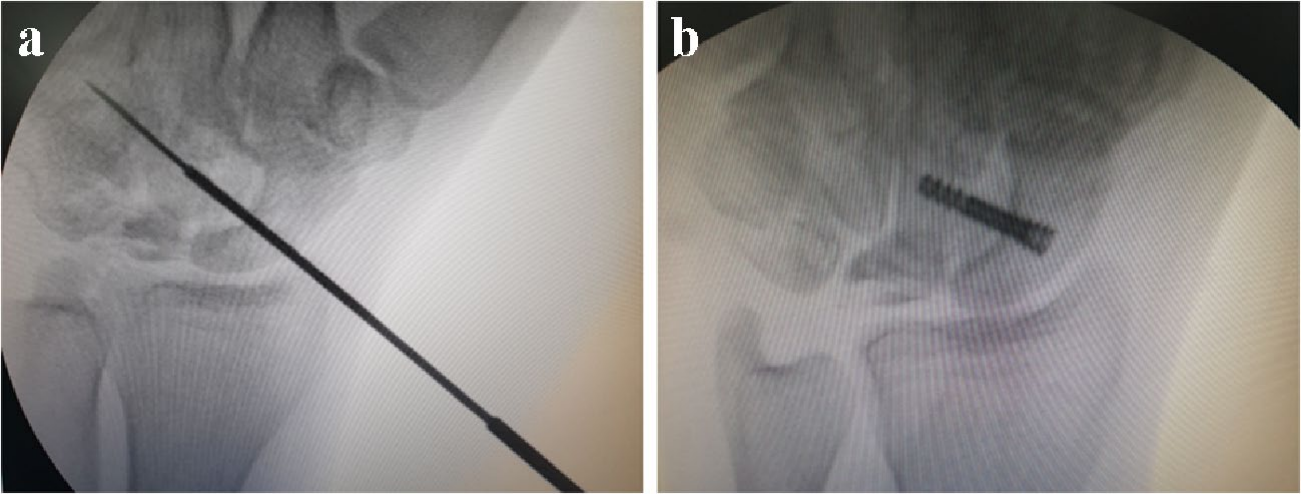
Intraoperative views of the wrist. **a**) Guide wire for the headless screw with the cannulated drill bit. **b**) Fixation with headless compression screw

The screw length was determined by a measuring device over the guide wire. The screw was inserted over the guide wire after pre-drilling the port of entrance (Fig. 4).

Finally, the incisions of the portals were dressed with gauze without sutures, and then sterile dressing was applied. The wrist is then immobilized with a short arm plaster cast for about three weeks.

## Results

### Evaluation

The minimum follow-up period was 24 months with a mean of 29.2 months (range from 24 to 37 months). Clinical and radiological evaluation was done by two authors. Clinical evaluation was performed using Mayo wrist score, which assesses four domains: pain, flexion – extension arc ROM, grip strength, and functional status (return to employment or activities and satisfaction) [12]. Radial and ulnar deviations were also evaluated. Goniometer was used to measure ROM; active flexion—extension arc and active radial and ulnar deviations as a percentage of the contralateral side, while grip strength was measured by hand dynamometer as a percentage of the contralateral side. Visual Analog Scale (VAS) was also used for pain assessment. Radiological evaluation was carried out by plain X-ray and computed tomography (CT) scan of the wrist. Radiological fusion was assessed by the presence of cortical continuity, bridging trabeculae, and progressive loss of the fusion line. Signs of osteoarthritis involving the radioscaphoid articulation were also noted on the radiographs.

### Statistical analysis

Data were fed to the computer and analyzed using IBM SPSS software package version 20.0. **(**Armonk, NY: IBM Corp**)**. Categorical data were represented as numbers and percentages. Quantitative data were expressed as a range (minimum and maximum), mean, standard deviation, median, and interquartile range (IQR) for normally distributed quantitative variables. Paired t-test was used to compare between two periods.

### Final Results

**Fig. 5:**
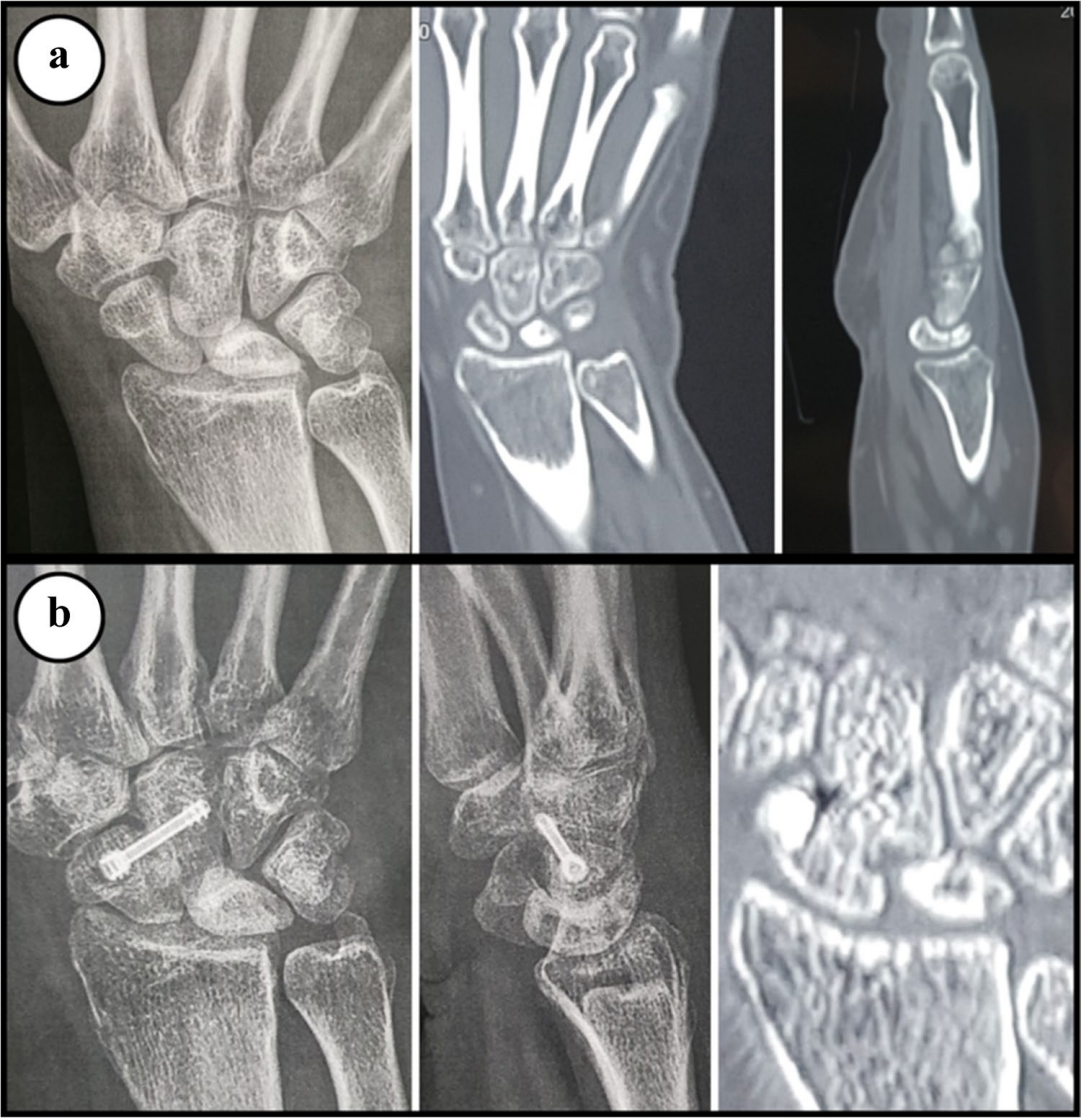
Example of radiographic images of a patient from this study. **a**) Pre-operative X-ray and CT scan. **b**) Post-operative X-ray and CT scan. Note that the trabeculae bridging the fusion line indicate fusion

There were 30 patients operated on in the study and no patient lost the follow-up. Fusion was achieved in 29 patients in an average of 7.2 weeks (range 6 to 10 weeks) (Fig. 5). In this study, there were no patients where arthroscopic surgery was changed to open surgery intraoperatively. Duration of operation was about three hours at the beginning of the study; however, with being familiar with the technique, it was reduced to about an hour to an hour and a half (Table 3).

**Table 3:** Final Results

	No. (%)
Operation time (minutes)	
Min. – Max	60 – 180
Mean ± SD	116.70 ± 36.04
Median (IQR)	110 (90 – 150)
Fusion status	
Fusion	29 (96.7%)
Non-fusion	1 (3.3%)
Fusion time (weeks)	
Min. – Max	6 – 10
Mean ± SD	7.21 ± 1.18
Median (IQR)	7 (6 – 8)
Follow-up period (months)	
Min. – Max	24 – 37
Mean ± SD	29.20 ± 4.60
Median (IQR)	27 (25 – 34)

SD: **Standard deviation**, IQR: **Inter quartile range**

Mayo wrist score revealed 26 satisfactory and four unsatisfactory results, with an average of 67.5 (range 30 to 85). Flexion – extension arc ROM increased from 68.17% to 70.83% of that of the contralateral wrist which was not a statistically significant difference. Radial deviation decreased from 64.22% to 57.47% and ulnar deviation decreased from 69.53% to 62.88% of that of the contralateral wrist which was a statistically significant difference. Grip strength increased from 40.21% to 70.20% of that of the contralateral side which was a statistically significant difference (p =  < 0.001). VAS significantly improved after surgery (Table 4). Most patients return to their work and daily activities.

**Table 4:** Pre-operative and post-operative flexion – extension arc ROM, radial deviation, ulnar deviation, grip strength, VAS, and Mayo wrist score

	Pre-operative Mean ± SD	Post-operative Mean ± SD	Test of significance	*p*-value
Flexion – extension ROM proportion to contralateral wrist (%)	68.17 ± 12.39	70.83 ± 11.49	t = 1.183	0.246
Radial deviation proportion to contralateral side (%)	64.22 ± 18.39	57.47 ± 14.53	t = 2.795^*^	0.009*
Ulnar deviation proportion to contralateral side (%)	69.53 ± 15.63	62.88 ± 16.21	t = 2.788^*^	0.009*
Grip strength proportion to contralateral side (%)	40.21 ± 15.64	70.20 ± 19.15	t = 8.959^*^	< 0.001^*^
VAS	6.33 ± 1.71	2.70 ± 2.04	Z = 4.463*	< 0.001^*^
Mayo wrist score	33.33 ± 11.47	67.50 ± 13.57	t = 13.115*	< 0.001^*^

SD: **Standard deviation**, **t: Paired t-test**, **Z: Wilcoxon signed ranks test**

*: Statistically significant at p ≤ 0.05

No wound infection, no neurovascular complications, and no implant failure were observed. No radioscaphoid arthritis was detected during the evaluation of pre-operative and post-operative radiographs of all patients. Only one patient had non-fusion, open bone graft was done and fusion was achieved. Another patient had a prominent screw, arthroscopy was done to evaluate the articular cartilage for any damage by the screw, however, no damage was observed, and so the screw was tightened till the head of the screw was sunken into the scaphoid.

## Discussion

This study validates the effectiveness of arthroscopic SCF in patients with stage IIIB and IIIC Kienböck’s disease with satisfactory clinical, radiological, and functional results without the need for bone graft; as fusion was achieved in an average of 7.2 weeks.

This minimally invasive surgery depends on combining the advantages of arthroscopy, dry technique, percutaneous fixation, and not requiring bone graft; all leads to the hoped-for outcome achieved by minimizing surgical insult to the wrist.

Arthroscopy has wide intra-articular exposure of the wrist, causing minimal damage to the ligaments and capsule thus preserving the blood supply to the carpals and preserving joint mobility which leads to more rapid healing [9]. The posterior interosseous nerve is also preserved and the proprioception of the joint is maintained, resulting in less arthritic changes and preferable healing [9, 13, 14].

Dry wrist arthroscopy was chosen in this study due to its obvious advantages of good clarity due to the index of reflection of the air, which is inside the joint, being very close to one. Accurate visualization can be lost because of turbulence in the saline [15]. Vertical traction provides the necessary joint distension without the need for saline distension; as the structure of the wrist joint allows for this [9]. The dry technique allows the operation to be performed without fluid extravasation and tension from water is not a concern. The dry technique allows longer procedures as bony landmarks are not blurred by the extravasated fluid, and avoids the complications of extravasated fluid [9].

Percutaneous fixation results in less soft tissue dissection, less disruption of vascularity, and shorter operative time [16]. Avoiding bone graft has the advantage of avoiding donor site morbidity, increased probability of infection, and chronic pain.

As far as we know, the number of articles on arthroscopic SCF is quite limited; moreover, not requiring bone graft results in even fewer articles, and this study is the largest prospective case series to date.

Nonunion has been reported from zero to 23% with open SCF and from zero to 10% with arthroscopic SCF [3, 17–21]. Similarly, in this study there was only one patient (3.3%) had non-fusion, knowing that he was a heavy smoker. The low incidence of nonunion and rapid fusion, achieved without bone graft, with arthroscopic surgery may be due to the preservation of the soft-tissue attachments and the blood supply to the carpals with a less invasive procedure.

The Lunate was not excised in this study. Rhee PC et al. described in a retrospective study that the presence or absence of the lunate did not significantly affect clinical outcomes in cases of limited carpal fusion for Kienbock’s disease [22]. Koh IH et al. reported that when comparing patients in whom the lunate was removed or not, the VAS and Modified Mayo Wrist Score (MMWS) were significantly improved in both cases, as was the case in this study. Hence, considering the added surgical time of lunate excision, it is thought that maintaining the lunate is preferable [23]

The pioneer of dry arthroscopy Francisco del Piñal presented a technique for dry arthroscopic scaphoidectomy and four-corner fusion. In their series, despite the first operation which lasted four hours, the last two operations were completed in less than two hours. No operations were converted to an open procedure [9]. Comparable to our results, halfway through this study the operative time was around one hour and 20 min, starting the study with an operative time of around three hours, and we did not convert any patient in this study to an open procedure.

Partial wrist fusions, including SCF, improve muscular strength by reducing pain after surgery and allow patients to return to their daily lives [24]. In this study, most patients showed improvements in terms of pain and grip strength. Flexion – extension arc ROM increased compared to the contralateral wrist, though this difference was not statistically significant, while radial and ulnar deviation decreased significantly compared to the contralateral wrist. Bouri et al. found similar results to this study regarding ROM in their systematic review and meta-analysis of the outcomes of open SCF in Kienböck’s disease, except for the ulnar deviation, where there was no significant difference after the surgery [25].

The literature primarily discusses open surgical techniques, meanwhile Leblebiciog˘lu et al., Baur, and Koh et al. performed SCF arthroscopically achieving generally good outcomes close to the result of this study [3, 4, 23]. Those studies used bone graft in contrast to Ertem et al. who did not use bone graft. Ertem in his study included 11 patients with Kienböck’s disease with a statistically significant difference in Mayo wrist score of post-operative third and sixth months and pre- and post-operative Quick Disabilities of Arm, Shoulder and Hand scores [19].

The most frequent complication of wrist arthroscopy was the failure to achieve the procedure (1.16%), and nerve lesions (1.17%) as stated by Leclercq C et al. Other complications include cartilage lesions, complex regional pain syndrome, wrist stiffness, and tendon lacerations with total complication incidence of wrist arthroscopy in about 5.98% of procedures done [26]. However, no complications described above were seen in this study.

This study has few limitations. The relatively short period for follow-up does not assess rates of delayed radiological and functional results such as long-term incidence of radioscaphoid arthritis. Thus, longer follow-up is needed. There also was no control group assessing the difference in outcomes compared to open SCF, so further prospective cohort studies are required. This study is meaningful in that it was done on a large number of patients to show that arthroscopic SCF can produce satisfactory results even without bone graft.

Arthroscopic SCF may be a quite challenging procedure initially, but we hope it will be standard practice, just like in our hospital, as it achieved significant improvement in pain and wrist function doing so with arthroscopy and without the need for bone graft or excision of the lunate.

## Conclusions

Our study demonstrates that arthroscopic SCF is a valuable technique and an appropriate method for managing Kienböck’s disease. Being minimally invasive, it achieved significant improvements in pain and wrist function and has satisfactory clinical, functional, and radiological outcomes with a high union rate and rare complications.

## Data Availability

The authors confirm that the data supporting the findings of this study are available from the corresponding author, upon reasonable request.
